# Reclassification of Seronegative Rheumatoid Arthritis as Anti-PL-12 Antisynthetase Syndrome with Interstitial Lung Disease and Joint Involvement–Case Report

**DOI:** 10.3390/reports8030123

**Published:** 2025-07-26

**Authors:** Diana Elena Cosău, Alexandru Dan Costache, Irina Iuliana Costache Enache, Ionela Lăcrămioara Șerban, Luiza Andreea Petrariu, Cristina Pomîrleanu, Mara Russu, Vladia Lăpuște, Codrina Ancuța

**Affiliations:** 1 Faculty of Medicine, “Grigore T. Popa” University of Medicine and Pharmacy, 700115 Iasi, Romania; cosau.diana-elena@d.umfiasi.ro (D.E.C.); irina.costache@umfiasi.ro (I.I.C.E.); ionela.serban@umfiasi.ro (I.L.Ș.); daniela.pomirleanu@umfiasi.ro (C.P.); mara.russu@umfiasi.ro (M.R.); codrina.ancuta@umfiasi.ro (C.A.); 2 Clinical Rehabilitation Hospital, 700661 Iasi, Romania; alpetrariu@yahoo.com; 3 “St. Spiridon” Emergency County Hospital, 700111 Iasi, Romania

**Keywords:** antisynthetase syndrome, PL-12 antibody, interstitial lung disease, rheumatoid arthritis, overlap syndrome, amyopathic myositis, Sjögren’s syndrome

## Abstract

**Background and Clinical Significance:** Antisynthetase syndrome (ASyS) is a rare autoimmune entity defined by the presence of anti-aminoacyl-t ribonucleic acid (RNA) synthetase autoantibodies and classically associated with a triad of interstitial lung disease (ILD), inflammatory myopathy, and arthritis. Additional clinical features may include Raynaud’s phenomenon and “mechanic’s hands”. Among antisynthetase antibodies, anti-PL-12 is notably associated with predominant or isolated ILD and may occur in the absence of clinically evident myositis, thereby complicating timely diagnosis. **Case Presentation:** We are presenting a 45-year-old non-smoking female patient with a prior diagnosis of seronegative rheumatoid arthritis (RA) who developed progressive dyspnea, dry cough, and sicca symptoms. High-resolution computed tomography revealed a nonspecific interstitial pneumonia (NSIP) pattern. Despite normal creatine kinase and lactate dehydrogenase levels, serological work-up revealed positive anti-PL-12 and anti-Ro52 antibodies, supporting a diagnosis of antisynthetase syndrome without myositis, fulfilling the diagnostic criteria for ASyS per Connors and Solomon. Treatment with corticosteroids and cyclophosphamide induced clinical and functional respiratory improvement, while azathioprine was initiated for maintenance. **Conclusions:** This case underscores the clinical heterogeneity of antisynthetase syndrome and highlights the diagnostic challenge posed by anti-PL-12–associated ILD in the absence of myositis. Importantly, it demonstrates that in patients with pre-existing rheumatologic diagnoses, the emergence of atypical pulmonary manifestations warrants repeat serologic evaluation to assess ASyS and other autoimmune conditions. Early diagnosis and immunosuppressive treatment are essential to optimize outcomes.

## 1. Introduction and Clinical Significance

Antisynthetase syndrome (ASyS) is a distinct autoimmune condition characterized by the presence of autoantibodies directed against aminoacyl-transfer ribonucleic acid RNA (tRNA) synthetases. Clinically, it is defined by a constellation of features that may include inflammatory myopathy, interstitial lung disease (ILD), Raynaud’s phenomenon, arthritis, fever, and the cutaneous sign known as “mechanic’s hands”. Among the inflammatory myopathies, ASyS is notably associated with a higher prevalence and severity of ILD compared to polymyositis or dermatomyositis, rendering pulmonary involvement a major determinant of prognosis and mortality [1].

Diagnosis of ASyS necessitates a multidisciplinary approach, incorporating rheumatologic and pulmonary evaluation, serological profiling (e.g., anti-Jo-1, anti-PL-7 [anti-threonyl], anti-PL-12 [anti-alanyl-tRNA synthetase] antibodies), high-resolution computed tomography (HRCT), and in selected cases, histopathological assessment via lung or muscle biopsy. The clinical heterogeneity of ASyS underscores the importance of early recognition, especially when myopathy is absent or subclinical [1].

Interstitial lung disease encompasses a broad spectrum of disorders with diverse etiologies and variable prognoses, unified by the pathological expansion of the pulmonary interstitium through inflammation and/or fibrosis. ILDs may be idiopathic or secondary to systemic diseases, including autoimmune rheumatic disorders, such as ASyS, systemic sclerosis, and rheumatoid arthritis (RA). When idiopathic causes are excluded, ILD in the setting of systemic autoimmunity may be classified as a manifestation of connective tissue disease (CTD-ILD), with ASyS representing a prototypical overlap [1].

Within the idiopathic inflammatory myopathies (IIMs), two major autoantibody-defined subgroups are frequently associated with ILD: antisynthetase syndrome and anti-MDA5 (melanoma differentiation-associated gene 5) myositis, each with distinct clinical and prognostic profiles. ASyS is often the predominant phenotype among IIMs with chronic ILD, whereas anti-MDA5 is typically linked to rapidly progressive ILD with poor prognosis [1].

Management of ASyS involves immunosuppressive therapy tailored to disease severity and organ involvement. Corticosteroids remain the first-line treatment during acute flares; however, long-term control frequently requires steroid-sparing agents, such as azathioprine, mycophenolate mofetil, cyclophosphamide, cyclosporine, tacrolimus, or biologics, including rituximab [1].

The connection between ASyS and RA is exceptionally uncommon [1].

## 2. Case Presentation

We report the case of a 45-year-old non-smoking female patient from a rural environment, with no documented exposure to inhaled toxins or occupational respiratory hazards, who was admitted to the Rheumatology Department of the Clinical Rehabilitation Hospital in Iași, Romania. She was referred for comprehensive evaluation of persistent fatigue, progressive exertional dyspnea (grade 2 on the modified Medical Research Council [mMRC] scale), a chronic dry irritative cough, and symmetrical inflammatory polyarthralgia predominantly involving the small joints of the hands; morning stiffness exceeded one hour. Additionally, the patient also reported sicca symptoms—dry eyes and mouth—that had emerged approximately four months prior to admission, along with constitutional symptoms, including weight loss, fatigue, and anorexia.

Her medical history was notable for a diagnosis of seronegative RA, established in April 2018, based on the presence of symmetrical inflammatory polyarthritis involving the small joints of the hands, prolonged morning stiffness exceeding one hour, and elevated inflammatory markers-C-Reactive protein (CRP) at 28 mg/L and erythrocyte sedimentation rate (ESR) of 52 mm per hour, in the absence of detectable rheumatoid factor (RF) or anticitrullinated protein antibody (ACPA). She had been treated with leflunomide, which initially provided good control of her articular symptoms. Extended myositis panels or antisynthetase antibodies were not checked, as no clinical signs of myositis or pulmonary symptoms (cough, dyspnea, Velcro-like crackles) were reported. Additional comorbidities included grade 3 essential hypertension with high cardiovascular risk, mixed dyslipidemia, sinus tachycardia, and major depressive disorder.

In June 2020, the patient was admitted to the Pneumology Department due to worsening respiratory symptoms, including dyspnea on minimal exertion, a persistent dry cough, and night sweats. Given the COVID-19 pandemic context, SARS-CoV-2 infection was initially suspected but ruled out by a negative PCR test. A high-resolution computed tomography (HRCT) scan of the chest revealed imaging features consistent with interstitial pulmonary fibrosis, confirming the diagnosis of ILD. At the time of admission, clinical evaluation and imaging were focused on the pulmonary symptoms. Although systemic features, such as fatigue, weight loss, anorexia, and night sweats, were already evident, they were initially attributed to constitutional or infectious etiologies. Sicca symptoms had begun to emerge but were not yet recognized as part of the systemic autoimmune process. There were no documented signs of Raynaud’s phenomenon or mechanic’s hands during the pneumology admission, and no autoimmune or myositis-specific serologic tests were performed. The clinical approach remained focused on excluding COVID-19 and managing what was presumed to be idiopathic or post-infectious ILD. The final diagnosis at discharge was idiopathic interstitial lung disease. The patient was prescribed symptomatic treatment, including inhaled bronchodilators and supportive respiratory care. No immunosuppressive therapy was initiated, as autoimmune etiology was not suspected at that time.

One month later, the patient was referred to the Rheumatology Department for further evaluation due to ongoing fatigue, constitutional symptoms, and the emergence of more overt systemic features. Notably, she reported experiencing Raynaud’s phenomenon affecting her fingers, with daily episodes triggered by cold or emotional stress. The episodes included pallor, cyanosis, and subsequent rubor, but there were no signs of digital ulcers or ischemia. Sicca symptoms also persisted; however, she did not report muscle weakness.

On clinical examination, the patient reported mild exertional dyspnea with a respiratory rate of 20 breaths per minute and peripheral oxygen saturation (SpO_2_) of 96% on room air. She was afebrile, with a body temperature of 36.0 °C. Chest auscultation revealed basal crackles audible over both lower pulmonary areas. The patient reported no history of smoking, recreational drug use, exposure to contagious areas, or recent travel.

Musculoskeletal examination demonstrated mild synovial swelling and tenderness involving the metacarpophalangeal (MCP), proximal interphalangeal (PIP), and radiocarpal joints bilaterally. She reported morning stiffness lasting over one hour daily. These findings contributed to a Disease Activity Score in 28 joints (DAS28) of 5.16, indicating high disease activity.

Dermatological examination revealed a characteristic scaly, fissured, hyperkeratotic erythematous eruption with an exfoliative appearance on the palmar surfaces and lateral aspects of the fingers and hands. This presentation was consistent with “mechanic’s hands”, a cutaneous hallmark of antisynthetase syndrome. No other dermatologic signs typically associated with dermatomyositis were observed, including Gottron’s papules or heliotrope rash. Additionally, clinical examination revealed Raynaud’s phenomenon affecting the fingers of both hands. A Raynaud Condition Score of 3/10 was recorded, based on daily episodes precipitated by cold exposure. Digital pitting scars were also noted, indicating chronic microvascular injury. There were no signs of digital ulcers or critical ischemia.

Laboratory evaluation revealed elevated markers of systemic inflammation, with a CRP level of 47 mg/L and ESR of 65 mm/h. Complete blood count showed mild leukocytosis (white blood cell = 10.600/L) and neutrophilia (neutrophils = 8510/L), likely secondary to corticosteroid exposure, as well as reactive thrombocytosis (platelets = 435,000/L) indicative of active inflammation. Renal, hepatic, and thyroid function panels were within normal ranges. Urinalysis showed no abnormalities.

Given the constellation of clinical findings, an idiopathic inflammatory myopathy (IIM) was suspected. However, serum muscle enzymes levels, including creatine kinase (CK: 5.48 U/L), and lactate dehydrogenase (LDH: 65 U/L), were within normal limits, raising the possibility of an amyopathic phenotype. Comprehensive infectious screening yielded negative results, including SARS-CoV-2, influenza, and respiratory syncytial virus, via rapid polymerase chain reaction (PCR), as well as negative serologies for hepatitis B surface antigen (HBsAg), hepatitis B surface antibody (HBsAb), and an unreactive tuberculosis (TB) spot test.

An extensive work-up was undertaken to elucidate the etiology of this interstitial pulmonary fibrosis. The immunological profile showed positive antinuclear antibodies (ANA) immunoglobulin G (IgG) subtype and the presence of Ro-52/SS-A (anti-Sjögren’s syndrome-related antigen A autoantibodies) consistent with a possible underlying autoimmune process. Additionally, the RF was elevated at 70 IU/mL while the ACPA was still negative. An extended myositis antibody panel demonstrated positivity for anti-PL-12 antibodies, a defining serological marker of ASyS; other myositis-specific and associated antibodies, including anti-Mi-2, anti-Ku, anti-polymyositis/Scleroderma 70 (PM-SCL 70), anti-centromere, anti-Jo-1, anti-signal recognition particle (SRP), anti-PL-7, and anti-Ej, were negative. Additional immunologic investigations were also unremarkable with negative results for antineutrophil cytoplasmic antibodies (ANCA), anti-double-stranded deoxyribonucleic acid (DNAdc), anti-Smith (Sm), anti-U1 small nuclear ribonucleoprotein particle (U1-RNP), and anti-Sjögren’s syndrome-related antigen B autoantibodies (LA/SS-B). The cumulative serologic profile, including isolated anti-PL-12 and anti-Ro52 positivity in the absence of myositis-specific enzyme elevation, supported the diagnosis of amyopathic antisynthetase syndrome with overlapping Sjögren’s features [see Table 1].

Pulmonary function testing (PFT) was conducted to assess the degree of functional impairment associated with ILD. Spirometry results were within normal range: forced vital capacity (FVC) was 90% of the predicted value, forced expiratory volume in 1 s (FEV1) was 95%, and the diffusing capacity of the lungs for carbon monoxide (DLCO) was 75% of the predicted value. Peripheral oxygen saturation (SpO_2_) was 96% at rest and increased to 97% following a 6-min walk test covering 400 m, suggesting preserved gas exchange during exertion. Despite these objective findings, the patient continued to report subjective breathlessness. This clinical–functional dissociation may reflect early interstitial involvement insufficient to produce significant physiological changes or may be multifactorial, potentially attributable to systemic inflammation, physical deconditioning, underlying cardiovascular comorbidities, or psychological factors, such as anxiety and depressive disorder.

**Figure 1:**
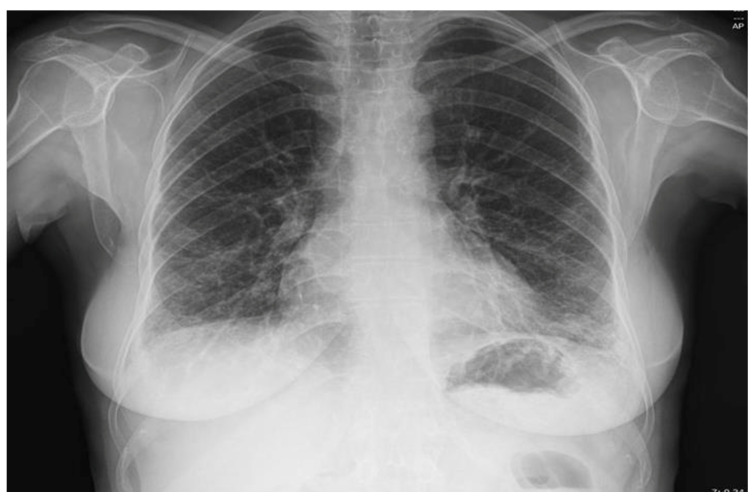
Chest X-ray showing a more accentuated bilaterally basal reticular lung pattern.

The electrocardiogram (ECG) indicated a sinus rhythm with a normal QRS width and no evidence of ischemic heart disease. Although transthoracic echocardiography revealed moderate left ventricular diastolic dysfunction and mild septal hypertrophy, there was no echocardiographic evidence of elevated pulmonary artery systolic pressure (PAPS) or right heart dysfunction. These findings, while not indicative of pulmonary hypertension, may suggest subclinical cardiac involvement contributing to exertional dyspnea. Importantly, biventricular systolic function remained intact. A baseline chest X-ray revealed a more pronounced bilateral basal reticular pattern (see Figure 1), consistent with interstitial changes.

**Figure 2:**
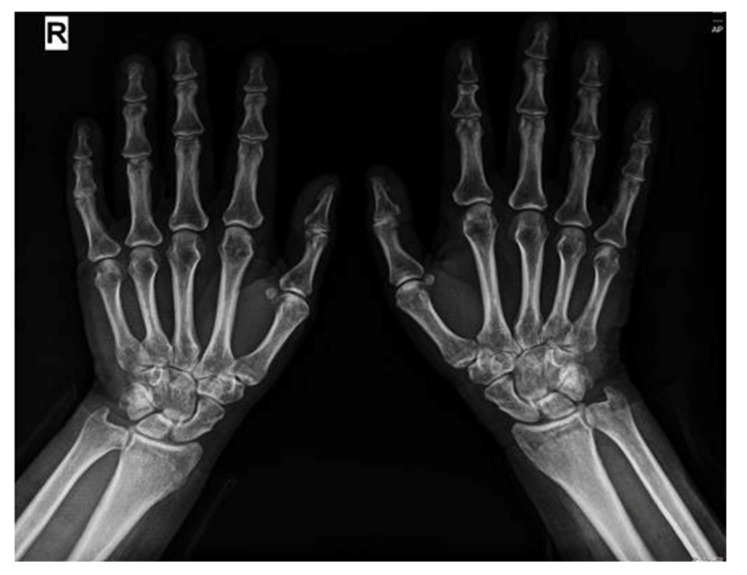
Hand X-ray showing non-erosive changes.

The hand X-ray shows right pisiform geode, minimal joint spaces at metacarpophalangeal levels III, IV and proximal interphalangeal levels IV, V bilaterally (see Figure 2).

A second HRCT scan of the chest was performed aiming to further characterize the ILD initially identified in June 2020, as the initial HRCT confirmed fibrosing interstitial changes, but it lacked sufficient detail to allow definitive radiologic subclassification.

**Figure 3:**
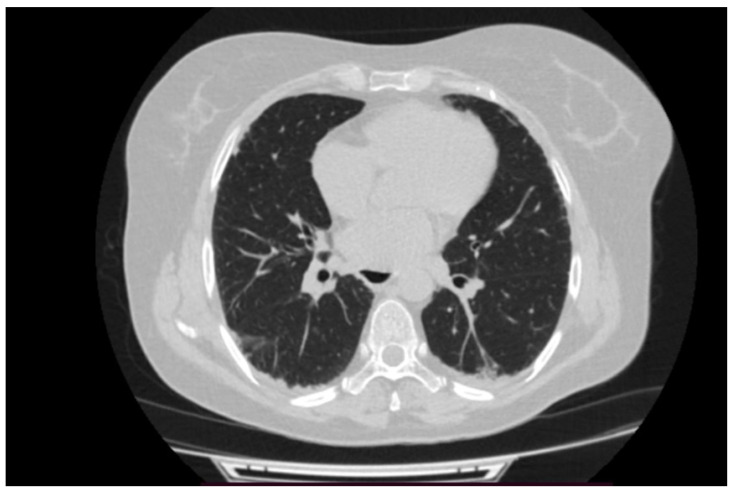
HRCT showing NSIP (transversal section).

**Figure 4:**
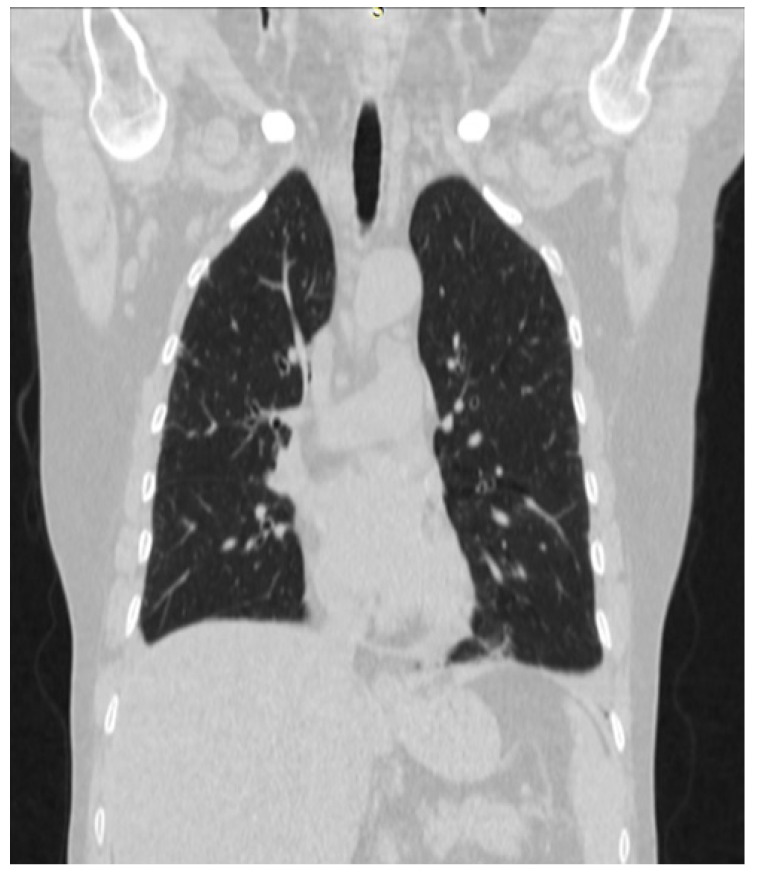
HRCT showing NSIP (frontal section).

The follow-up HRCT enabled a more accurate characterization of the interstitial process. The scan revealed mild to moderate fibrotic changes predominantly affecting the basal regions of both lungs, accompanied by scattered ground-glass opacities and limited traction bronchiectasis. (see Figure 3 and Figure 4). These findings were consistent with a nonspecific interstitial pneumonia (NSIP) pattern (see Figure 3 and Figure 4). Only the second HRCT was available for documentation and detailed radiological review.

A later CT scan of the abdomen and pelvis revealed no significant abnormalities. However, a moderate gastro-esophageal reflux was identified during a barium transit examination.

The constellation of clinical manifestations, including Raynaud’s phenomenon, “mechanic’s hands”, and non-erosive small joints arthritis, in conjunction with paraclinical findings, supported a unifying autoimmune diagnosis. Specifically, the presence of systemic inflammation, amyopathic myositis (characterized by normal CK and LDH levels), interstitial pulmonary fibrosis with mildly reduced DLCO confirmed by HRCT, and a distinct serologic profile—positive anti-Ro/SSA, anti-Ro 52, and anti-Pl 12 antibodies—established the diagnosis of anti-PL-12-positive ASyS presenting with ILD and arthritis, in the absence of overt myositis.

**Figure 5:**
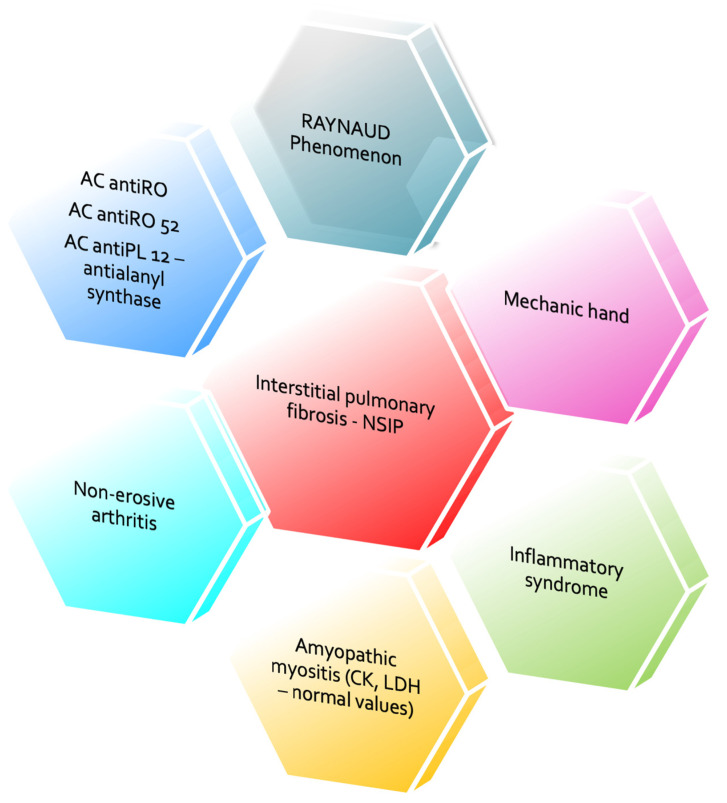
Integration of clinical and paraclinical data.

This integrative reassessment led to the reclassification of the previously presumed diagnosis of seronegative RA as a manifestation of ASyS. The presence of sicca symptoms and anti-SSA positivity further supported the diagnosis of an overlapping Sjögren’s disease (see Figure 5).

## 3. Treatment and Outcome

Since interstitial pulmonary fibrosis is the one that dictates the prognosis in ASyS, immunosuppressive treatment was initiated promptly. The patient received 6 monthly cycles of intravenous Cyclophosphamide administrated as pulse therapy at a dose of 600 mg per cycle, diluted in 250 mL of physiological saline. Concomitantly, oral methylprednisolone was started at 0.5 mg/kg/day, along with Pantoprazole 40 mg/day therapy for gastric protection. To maintain remission, Azathioprine was introduced gradually: 50 mg/day for the first week, then 50 mg bis in die (bid) in the second week, and finally 50 mg ter in die (tid) as a long-term maintenance dose. Background therapy for associated comorbidities (hypertension, dyslipidemia, and depression) was continued. The medication for the associated conditions was also added to the treatment schedule.

Following the initiation of corticosteroid and cyclophosphamide therapy, the patient reported subjective improvement in dyspnea, myalgia, and arthralgia, with full resolution of the “mechanic’s hands”. Inflammatory markers decreased, and muscle enzymes remained within normal range. One year later, repeated pulmonary function tests showed a mild restrictive ventilatory defect (DLCO 69% of the predicted value, FVC = 76% of the predicted value, FEV1 was 77%), indicating a modest decline from initial baseline values. Despite this decline, the patient’s respiratory symptoms were perceived as improved, possibly due to a better systemic disease control, reduced inflammatory burden, or improved physical conditioning. Maintenance therapy with Azathioprine 50 mg tid was continued. The dose of methylprednisolone was tapered progressively over 12 months, currently maintained at a minimal effective dose of 4 mg/day.

Regarding the prognosis, initiation of treatment less than 6 months after the diagnosis was established, prompt response to corticosteroid and immunosuppressive therapy, age < 60 years, female gender, and the non-smoker status suggest a favorable outcome of the disease.

## 4. Discussion

The diagnostic criteria suggested by Connors et al. and Solomon et al. enable the recognition of ASyS as a distinct condition. This distinction can prove valuable in both clinical practice and research context [2] (See Table 2).

Connors et al. and Solomon et al. have put forth diagnostic criteria for antisynthetase syndrome. Connors et al. stipulate that the presence of an anti-aminoacyl tRNA synthetase antibody is essential, along with one or more clinical features, such as arthritis, Raynaud’s phenomenon, ILD, mechanic’s hands, and fever [3].

On the other hand, Solomon et al. require the presence of an anti-aminoacyl tRNA synthetase antibody along with either two major criteria or one major criterion and two minor criteria. The major criteria consist of ILD and dermatomyositis or polymyositis, while the minor criteria include Raynaud’s phenomenon, arthritis, and Raynaud’s phenomenon [3]. The patient in this case met the criteria for the diagnosis of antisynthetase syndrome proposed by both Connors et al. and Solomon et al. (see Table 2) [4].

Our patient was diagnosed with ASyS due to the presence of polymyositis, arthritis, a positive anti-PL 12 antibody, mechanic’s hands, and adherence to the criteria established by both Connors et al. and Solomon et al. These findings are consistent with the updated European criteria proposed by the American-European Consensus Group [5].

### 4.1. Differential Diagnosis

Given the patient’s prior diagnosis of seronegative RA-associated interstitial lung disease (RA-ILD), this entity represented a relevant differential diagnosis during the initial pulmonary evaluation. RA-ILD typically presents with a NSIP or usual interstitial pneumonia (UIP) pattern and is often associated with ACPA and/or RF positivity. However, it is increasingly recognized that seronegative RA may also be complicated by ILD, albeit less frequently and with more diagnostic ambiguity. Recent studies describe seronegative RA as a clinical form characterized by classical symptoms, such as joint stiffness and swelling and elevated inflammatory markers, but lacking anti-CCP and RF antibodies. In this case, the absence of ACPA and the presence of evolving systemic features, such as Raynaud’s phenomenon, sicca symptoms, and “mechanic’s hands”, along with a positive anti-PL-12 antibody, supported an alternative diagnosis of ASyS. Nonetheless, RA-ILD remained a plausible initial consideration, particularly in the context of persistent respiratory symptoms and a history of inflammatory arthritis. This overlap highlights the importance of comprehensive autoantibody profiling and HRCT in distinguishing ASyS from other connective tissue disease-associated ILDs, including those seen in RA [6].

Another differential diagnosis considered was systemic sclerosis (SSc) sine scleroderma—a rare subset of systemic sclerosis that manifests with visceral organ involvement (ILD, esophageal dysmotility) in the absence of cutaneous sclerosis. Although the patient lacked skin thickening, digital ulcers, and tested negative for anti-Scl-70 and anti-centromere antibodies, the constellation of ILD and Raynaud’s phenomenon warranted consideration of this entity. However, the absence of esophageal symptoms and negative serological markers made this diagnosis unlikely [7].

SSc was also excluded due to the absence of cutaneous manifestations (sclerodactyly, digital ulcers) and negative immunologic markers (anti Scl-70, anti-cetromere, PM-Scl antibody) [7].

Systemic lupus erythematous was excluded based on negative serologic markers anti-DNAdc and anti-Sm, as well as the absence of hematologic abnormalities (cytopenia) or renal involvement (proteinuria, hematuria) [8].

Mixed connective tissue disease was ruled out due to the absence of the U1RNP antibody negative) and the lack of clinical features suggestive of overlap syndromes involving systemic sclerosis, polymyositis, or lupus [9] (see Table 3).

In our case, the patient diagnosed with antisynthetase syndrome and associated ILD, along with positive anti-PL 12 antibodies, exhibited an unusual absence of concurrent myositis. This rarity in antisynthetase syndrome characterized by anti-PL 12 antibodies is underscored by the limited number of reported cases in the literature [5].

Once more, as outlined in this case, it is important to note that not all individuals with idiopathic IIM exhibit elevated muscle enzymes during initial presentation. In cases of ASyS-related ILD, HRCT of the chest frequently indicates NSIP, organizing pneumonia, or a combination of both [5]. This observation is consistent with recent data by Indu et al. (2025), who reported that HRCT revealed an NSIP pattern in 82% of patients with ASyS. These findings confirm the value of high-resolution imaging in the early differentiation of ILD subtypes in ASyS [10].

The patient in question exhibited only pulmonary involvement at the time of diagnosis, a characteristic often seen in individuals with a positive anti-PL12 antibody [11].

ASyS, a rare autoimmune condition, is often categorized as a distinct subtype of idiopathic inflammatory myopathy. It is marked by the presence of myositis, ILD, and arthritis, along with the existence of antibodies directed against aminoacyl-tRNA synthetases. Additional clinical manifestations may involve mechanic’s hands, Raynaud’s phenomenon, and calcinosis [5].

ASyS associated with ILD is generally considered to have poor prognosis; the presence of antisynthetase autoantibodies remains the most reliable predictor for the development of ILD in this context [12]. Pulmonary involvement is observed in approximately 70–100% of individuals with ASyS and mortality related to connective tissue disease-associated pulmonary fibrosis is estimated at around 20%. Corticosteroid therapy plays a central role in initial disease management [13].

The clinical presentation of idiopathic IIM is highly heterogeneous. Not all patients display muscle weakness or elevated CK levels at disease onset; these features may emerge later in the disease course. Therefore, in the presence of ILD—particularly when infectious etiologies have been excluded—a thorough evaluation for non-infectious, autoimmune causes is essential [13].

Treatment typically involves immunosuppression, where corticosteroids constitute the primary therapeutic approach. Early initiation of disease-modifying antirheumatic drugs (DMARDs) and the utilization of cyclophosphamide may be warranted in cases of life-threatening pathologies [14].

This case also highlights how systemic autoimmune inflammation, including forms initially labeled as seronegative RA, may contribute to early endothelial dysfunction. The combination of Raynaud’s phenomenon, Sicca symptoms, and ILD in our patient may reflect underlying microvascular injury driven by oxidative stress-a mechanism increasingly recognized in autoimmune connective tissue diseases. This observation aligns with current research directions focused on oxidative stress and vascular biomarkers in autoimmune arthritis [15].

In individuals with idiopathic IIM, various myositis-specific and associated autoantibodies have been identified and are routinely assessed during the investigation of idiopathic ILD (see Table 1 and Table 2). The distinctive feature of antisynthetase syndrome is the presence of myositis-specific antisynthetase antibodies [13].

### 4.2. Early Pulmonary Involvement in Antisynthetase Syndrome—Diagnostic and Prognostic Value

ASyS frequently manifests with respiratory symptoms, particularly ILD, which can serve as the initial or even the only symptom, preceding other systemic manifestations, such as myositis, arthritis, or Raynaud’s phenomenon. In this case, the respiratory symptomatology was the primary reason for medical presentation, subsequently directing investigations towards an autoimmune etiology and leading to the diagnosis of SSA. This clinical course underscores the significance of pulmonary involvement as a pivotal component in the early identification of the disease [16].

This observation is supported by recent data from a multicenter study (Freund et al., 2025), which reported that ILD was the initial clinical manifestation in 64% of ASyS cases. These results emphasize the need to include autoimmune etiologies in the differential diagnosis of ILD, especially in the absence of an obvious causative factor or in the presence of discrete rheumatologic features. The early detection of ILD as a potential sentinel manifestation of ASyS is essential for the prompt institution of immunosuppressive therapy and prevention of progression to irreversible lung damage [16].

Beyond its diagnostic implications, the autoantibody profile also appears to hold prognostic value. The same study showed an association between the presence of anti-Ro52 antibodies and a significant improvement in DLCO under treatment. This is consistent with the favorable course observed in our patient, who was anti-Ro52 positive and responded well to corticosteroid and cyclophosphamide, with subsequent maintenance on azathioprine [16].

These favorable factors are particularly relevant in anti-PL-12 positive cases. According to Zhao et al. (2022), early initiation of immunosuppressive therapy is essential in ASyS-ILD, especially in patients with anti-PL-12 antibodies, to prevent pulmonary function decline [17].

These data emphasize the importance of early pulmonary function assessment and comprehensive serological evaluation in patients with unexplained ILD. Furthermore, the identification of specific autoantibodies, such as anti-Ro52, may provide relevant information on therapeutic response and long-term prognosis. Recognizing these serological and clinical markers early in the disease course is crucial for optimizing patient outcomes and preventing irreversible pulmonary damage [16].

## 5. Conclusions

This case underscores the diagnostic complexity of ASyS, particularly in patients presenting with isolated ILD and in the absence of clinical evident myositis. The identification of anti-PL-12 and anti-Ro52 antibodies, alongside characteristic features, such as Raynaud’s phenomenon and “mechanic’s hands”, enabled the reclassification of previously presumed seronegative RA into a distinct amyopathic ASyS phenotype.

Importantly, this case highlights the need to reconsider autoantibody profiling in patients with established rheumatologic diagnoses who develop unexplained or atypical pulmonary manifestations. Extended serologic testing, including comprehensive myositis panels, can uncover evolving or overlapping autoimmune syndromes, such as ASyS, which may be overlooked in the absence of muscle enzyme elevation or overt myopathy.

Timely recognition and early initiation of immunosuppressive therapy, before the onset of irreversible pulmonary fibrosis are essential to improving long-term outcomes. This case illustrates the critical role of multidisciplinary collaboration between rheumatology and pneumology specialists in evaluating atypical autoimmune presentations. Clinicians should maintain a high index of suspicion for ASyS in the differential diagnosis of ILD, particularly when supported by subtle clinical signs and targeted serologic markers, to ensure accurate diagnosis, early intervention, and optimized disease management.

## Figures and Tables

**Table 1:** Myositis-specific and -associated antibodies (including antisynthetase antibodies).

Antibody Type	Serology	Results
	Anti-PL17	Negative
	Anti-PL12	Positive
Anty-synthetase	Anti-JO1	Negative
	Anti-EJ	Negative
	Anti-SRP	Negative
Myositis-specific	Anti-Mi-2	Negative
	Anti-MDA-5	Negative
	ANA	Positive
	Anti-RO/SSA	Positive
Myositis-associated	Anti-LA/SSB	Negative
	Anti-Ku	Negative
	Anti-U1 RNP	Negative
	Anti-PM-SCL	Negative

**Table 2:** The diagnostic criteria from Connors et al. and Solomon et al. [3].

Connors et al. (2010)	Solomon et al. (2011)
**Required:** Presence of an anti-aminoacyl tRNA synthetase antibody **PLUS** the presence of *one or more* specific clinical features, including: Raynaud’s phenomenon Arthritis Interstitial lung disease Unexplained fever Mechanic’s hands characterized by thickened and cracked skin on hands, especially at fingertips)	**Required:** Presence of anti-aminoacyl tRNA synthetase antibody **PLUS** the presence of either *two major criteria* or *one major criterion* and *two minor criteria*; Major: ILD (not attributable to another cause) Dermatomyositis or polymyositis based on Bohan and Peter criteria Minor: Raynaud’s phenomenon Arthritis Mechanic’s hands

**Table 3:** Differential diagnosis [6,7,8,9].

1. RA-ILD	-ACPA Negative
2. SSc sine scleroderma	-No cutaneous signs -No esophageal dysmotility (dysphagia absent) -Negative serology (anti-Scl70, anti-centromere, PM-SCL)
3. Systemic scleroderma	-Absent skin sclerosis (no sclerodactyly or digital ulcers) -Anti SCL-70 negative -Anti-centromere negative -PM-Scl negative
4. Mixed connective tissue disease	-Anti U1-RNP negative
5. Systemic lupus erythematosus	-Anti-DNAdc negative -Ac anti Sm negative -C3, C4 normal (no complement consumption) -no cytopenia

## Data Availability

The original data presented in this study are available on reasonable request from the corresponding author. The data are not publicly available due to privacy concerns.

## Abbreviations

The following abbreviations are used in this manuscript:

ACPA	Anti-citrullinated protein antibodies
ANA	Antinuclear antibodies
ANCA	Anti-neutrophil cytoplasmic antibodies
ANTI-SSA (Ro/52)	Anti-Sjögren’s-syndrome-related antigen A antibodies
ANTI-SSB (La)	Anti-Sjögren’s-syndrome-related antigen B antibodies
ASyS	Antisynthetase syndrome
BID	Bis in die
CK	Creatine Kinase
CT	Computed tomography
DAS28	Disease Activity Score 28
DLCO DMARDs	Diffusing capacity of the lung for carbon monoxide Disease-modifying antirheumatic drugs
DNA	Deoxyribonucleic acid
DNA-dc	Anti-double-stranded DNA antibodies
ECG	Electrocardiogram
ESR	Erythrocyte sedimentation rate
FEV1	Forced expiratory volume in 1 s
FVC	Forced vital capacity
HBsAb	Hepatitis B surface antibody
HBsAg	Hepatitis B surface antigen
HRCT	High-resolution computed tomography
IgG	Immunoglobulin G
IIM	Idiopathic inflammatory myopathy
ILD	Interstitial lung disease
LDH	Lactate dehydrogenase
MCTD	Mixed connective tissue disease
mMRC	Modified Medical Research Council (dyspnea scale)
NSIP	Nonspecific interstitial pneumonia
PAPS PCR	Pulmonary artery systolic pressure C-Reactive protein
PFT	Pulmonary Function Testing
PL-7	threonyl
PL-12	Anti-alanyl-tRNA synthetase
PM-Scl 70	Polymyositis/Scleroderma 70
RA	Rheumatoid arthritis
RF	Rheumatoid factor
RNA	Ribonucleic acid
Sm	Smith antigen (antibody)
SpO_2_	Peripheral oxygen saturation
SSc	Systemic sclerosis
TB	Tuberculosis
Tid	Ter in die
U1-RNP	U1 small nuclear ribonucleoprotein particle
UIP	Usual interstitial pneumonia
